# Preparing Dispersion Model Surface Meteorological Inputs Using High-Resolution Rapid Refresh (HRRR) Data

**DOI:** 10.21203/rs.3.rs-7557276/v1

**Published:** 2025-09-09

**Authors:** Xueying Zhang, Elaine Symanski, Hannah Renee Paduch, Itai Kloog, Yuxuan Wang, Yang Liu

**Affiliations:** 1. Department of Medicine, Section of Epidemiology and Population Sciences, Baylor College of Medicine, Houston, TX, USA; 2. Center for Precision Environmental Health, Baylor College of Medicine, Houston, TX, USA; 3. Department of Environmental Medicine, Icahn School of Medicine at Mount Sinai, New York, NY, USA; 4. Institute for Exposomic Research, Icahn School of Medicine at Mount Sinai, New York, NY, USA; 5. Department of Earth and Atmospheric Sciences, University of Houston, Houston, TX, USA; 6. Gangarosa Department of Environmental Health, Rollins School of Public Health, Emory University, Atlanta, Georgia, USA

**Keywords:** Dispersion model, HRRR, meteorologic data, air pollution

## Abstract

Accurate meteorological inputs are essential for air pollution dispersion modeling. Traditionally, dispersion models rely on observational meteorological data collected from weather stations at fixed locations. However, the sparse distribution of weather stations limits the ability to capture fine-scale meteorological variability, particularly in areas far from weather stations. In this study, we developed a novel framework for generating American Meteorological Society/Environmental Protection Agency (EPA) Regulatory Model (AERMOD) compatible surface meteorology data (.sfc) using the High-Resolution Rapid Refresh (HRRR) dataset, which provides predicted meteorological variables at a 3-km spatial resolution and an hourly temporal resolution. We followed the AERMOD Model Formulation document to create three scenarios of surface meteorology data, including two exploratory scenarios by setting HRRR meteorology parameter ranges and recalculating key parameters based on whether an hour filled in convective or stable planetary boundary layer status. We then applied these HRRR-derived meteorology data in the Research LINE source (R-LINE) dispersion model to predict traffic-related nitrogen dioxide (NO_2_) concentrations at 443 Air Quality System monitoring sites across the United States (U.S.) in the year 2019. For comparison, we also ran R-LINE using observational-based surface meteorology data preprocessed with AERMET. NO_2_ concentrations predicted by R-LINE were compared against NO_2_ measurement data by the meteorology inputs (three HRRR scenarios versus weather station data) using simple linear regression coefficient of determination (R^2^) and Index of Agreement (IOA). The three HRRR scenarios yielded a higher R^2^ on average (0.26) than did the observational data (R^2^=0.16), suggesting over 60% increase in explained variance. In simple linear regression analyses stratified by distance between NO_2_ sites and weather stations as well as by traffic magnitude around NO_2_ sites, HRRR data generally outperformed observational data. Site-specific IOA analyses further showed that, compared to observational meteorology data, HRRR inputs performed better across most of the continental U.S. but not as well in urban areas. Overall, our findings demonstrate that HRRR data has the potential to be utilized in air pollution dispersion modeling and that it has superior predictive ability in locations that lack nearby weather stations.

## Introduction

Dispersion models comprise a group of air pollution modeling tools used to estimate pollutant concentrations at specific locations based on emissions from sources and transport processes under varying meteorological conditions. One well-known dispersion model is the American Meteorological Society/Environmental Protection Agency (EPA) Regulatory Model (AERMOD), which is a designated model for multiple regulatory uses, including air permitting, state implementation plans, and facility-specific risk assessments. In addition to regulatory use, dispersion model outputs are increasingly utilized in air pollution research. For example, either AERMOD or the Research Line-source (R-LINE)^1^ dispersion models have been combined with land use regression or other geospatial models to predict ambient concentrations of air pollution, including particulate matters less than 2.5 microns aerodynamic diameter (PM_2.5_)^2, 3^, nitrogen dioxide (NO_2_),^4–6^ and air toxics.^3, 7^ And those studies showed that the high spatial and temporal resolution of dispersion models have the potential to increase overall modeling performance and downscale prediction metrics.

The quality of meteorological input is of great importance to dispersion model’s predictions. Such meteorology inputs are typically categorized as either “surface” variables, which represent conditions near the ground, or “vertical profile” variables, which represent features of the vertical structure of the atmosphere. Of these, the former are more widely used as they directly influence pollutant dispersion near the Earth’s surface. Key surface variables include wind speed, wind direction, temperature, and derived variables such as roughness, length, friction velocity (u*), and sensible heat flux (H). Accurate inputs of these variables are critical for the predictive reliability of dispersion models.

In dispersion models, the most widely used meteorological data are the observational data measured by weather stations, which are typically located in large airports. Several issues exist with employing this type of meteorological data in dispersion modeling. First, a given prediction location is typically assigned with meteorological data from the nearest weather station. However, weather stations are sparsely distributed geographically, with most counties in the U.S., for example, have only one or two stations. Therefore, meteorological data measured at weather stations does not accurately reflect local conditions at a distant site. In our previous work using the R-LINE dispersion model^1^ as a main predictor for modeling NO_2_ concentrations in the northeastern U.S.^6^, the distance between a predicted location (NO_2_ monitoring site) and its nearest weather station ranged from two hundred meters to 60 kilometers (km). And we observed that the overall predictive performance declined significantly when the nearest station was located more than 10 km from a NO_2_ monitoring site.

Several studies have explored possible substitutes for observational meteorological data in dispersion modeling to improve models’ performance. Modeled meteorological data have been considered because they provide spatially resolved, continuous coverage that made meteorological inputs in close proximity to prediction locations. Isakov et al.^8^ evaluated the performance of AERMOD in predicting tracer concentrations at one study site during a Tracer Field Study. They tested four different sources for AERMOD meteorological inputs, two observation-based and two modeled-based. The observational sources included local measurements and nearby airport’s National Weather Service measurements. The two model-based sources were spatially resolved meteorological data from a 12-km Eta Model and a 3-km PSU/NCAR mesoscale (known as MM5) prognostic model. When comparing coefficients of determination (R^2^) of linear regression models fitted with predicted and measured tracers, each meteorological input suggested comparable performance across observational sources (onsite measurement average R^2^ = 0.90; airport measurement R^2^ = 0.88) and modeled data (Eta model average R^2^ = 0.89; MM5 average R^2^ = 0.85).

The EPA has published guidelines on converting prognostic meteorological data from the Weather Research and Forecasting (WRF) model into AERMOD-ready format, same as the outputs of AERMET, AERMOD’s meteorology preprocessor. Studies^9–13^ have compared WRF-AERMOD predictions against observed air pollutant concentrations. However, due to the limited number of validation points in many of these studies, it is difficult to assess the accuracy of predictions from WRF-AERMOD. Similar to AERMOD, the R-LINE model uses the AERMET’s surface meteorology data. Parvez and Wagstrom^14^ examined diurnal and seasonal patterns in R-LINE predictions using WRF-derived surface meteorology compared to airport-based observations. They found that diurnal patterns of R-LINE predictions were similar across both data sources, while seasonal patterns from WRF-based inputs tended to show lower predicted values. However, because their predictions were traffic index values and not comparable to air pollution measurements, it was unclear whether WRF-derived meteorology could improve performance over observational meteorology data in R-LINE modeling.

Additionally, the complexity of operating AERMET has limited the widespread use of dispersion models that rely on it (e.g., AERMOD and R-LINE). AERMET requires input from multiple data sources and involves multiple steps and sub-processors with dependencies between stages. As a result, operating AERMET is often not user-friendly. Touma et al.^15^ demonstrated the feasibility of directly using meteorological data from the MM5 prognostic model as an input for AERMOD. For variables required by AERMOD but not directly available from MM5, the authors showed that they could be derived using equations provided in the AERMOD Model Formulation Document.^16^ As AERMOD and R-LINE are increasingly used in air permitting, exposure assessment,^4, 5, 17, 18^ and health studies,^19^ there is a growing need for more feasible approaches to prepare surface meteorological inputs for these models.

In this study, we propose a new approach to generate AERMET-format surface meteorological inputs. We utilized the National Oceanic and Atmospheric Administration’s High-Resolution Rapid Refresh (HRRR) dataset, which provides meteorological data at a 3-km spatial resolution and an hourly temporal resolution. For surface variables not directly available in HRRR, we calculated them following methods described in the AERMOD Model Formulation Document.^16^ We generated three HRRR preparation scenarios: (1) direct use of HRRR variables and derived unavailable variables with calculations in the AERMOD Model Formulation Document,^16^ (2) in addition to inputs for scenario (1), set upper and lower bounds for surface variables as defined in manual,^16^ and (3) in addition to inputs for scenario (2), perform separate calculations for the convective boundary layer (CBL) and stable boundary layer (SBL), consistent with the manual.^16^

To compare the performance of using HRRR-based data and observational meteorological data as inputs of dispersion model, we conducted an analysis using R-LINE. We obtained the AERMET data prepared with observational meteorology data for weather stations across US. The four versions of meteorology data, including the three scenarios of HRRR and the observation-based AERMET data, were used to run the R-LINE models to estimate traffic-related NO_2_ concentrations at 446 EPA Air Quality System (AQS) monitoring sites in 2019. We assessed model performance by comparing estimated NO_2_ concentrations against observed NO_2_ measurements at these sites, stratified by the meteorological data used.

## Methods

### Preparing AERMET-format surface meteorology data using HRRR

We downloaded the hourly surface meteorological data (wrfsfc) from the HRRR archive via Amazon Web Services (AWS). Data were downloaded for 2019 and both the zero-hour forecast (f00) and one-hour forecast (f01) products were obtained. We prepared all meteorology variables, except for the precipitation variable, using the f00 product. As all precipitation values are set to zero in the f00 data whereas f01 includes forecasted rainfall, the f01 product was appropriate for the precipitation variable. Although precipitation is not used by the R-LINE model in estimating dispersion effects, we still included the 1-hour forecasted precipitation as this variable may be used by AERMOD during certain modes.

The HRRR dataset contained a small number of missing hourly files. In 2019, the f00 product and f01 product were each missing seven hours. Missing data were first replaced using the prior hour’s forecast. If the prior hour was also unavailable, we substituted the previous hour’s forecast data.

Several variables in AERMET require different calculation methods depending on whether hours are classified as CBL or SBL. AERMET designates daytime as the CBL and nighttime as the SBL, and the transition between daytime and nighttime is determined by the hourly solar angle derived from meteorological observations. Because the current version of HRRR does not include solar angle, we used the Visible Beam Downward Solar Flux (variable name: “SFC = Ground or water surface; Visible Beam Downward Solar Flux [W/m^2^]”) as a proxy indicator. This variable generally has a value of zero during nighttime hours and positive values during the daytime hours. Below is a summary of how each AERMET surface variable was derived or calculated using HRRR data in Scenario 1:

- Year: study year in 2 digits, e.g., 2019 as 19.
- Month: study month, ranges from 1 to 12.
- Day of month: day of month, ranges from 1 to 31.
- Julian day: day of year, ranges from 1 to 366.
- Hour: hour of day, ranges from 1 to 24.
- Sensible heat flux, H: used HRRR variable “SFC=Ground or water surface; Sensible heat net flux [W/(m^2)]”.
- Surface friction velocity, u*: used HRRR variable “SFC=Ground or water surface; Frictional velocity [m/s]”.
- Convective velocity scale, w*: calculated using a formula^20^ from the AERMOD Model Formulation Document^16^:
$$w*={\left(\frac{g\;HZ_{\text{ic}}}{\rho \;c_{p}T_{\text{ref}}}\right)}^{1/3}$$
  where *g* is the acceleration due to gravity (9.8 m/s), *H* is the Sensible heat flux, *Z*_*ic*_ is the convective mixing height [using the HRRR planet boundary layer variable “SFC=Ground or water surface; Planetary boundary layer height [m]” as a proxy due to mixing height not being available in HRRR], *ρ* is the density of air [used as the dry air density (1.293 kg/m^3^) at a temperature of 273 Kelvin and a pressure of 101.325 kPa], *c*_*p*_ is the specific heat of air at constant pressure (1004 J/g/K), and *T*_*ref*_ is the ambient temperature representative of the reference surface layer [as the 2-meter temperature HRRR variable named “2[m] HTGL=Specified height level above ground; Temperature [C]” converted to Kevin].
- Vertical potential temperature gradient in the 500-m layer above the planetary boundary layer: as we were unable to find a suitable proxy for this variable in the HRRR data due to its not being utilized in R-LINE, we set this value to −9 as an indicator of missing data for this variable in AERMET.
- Convective mixing height, Z_ic_: used HRRR planet boundary layer as a proxy.
- Mechanical mixing height, Z_im_: used HRRR geopotential height variable, “EHLT=Equilibrium level; Geopotential height [gpm]” as a proxy.
- Monin-Obukhov length, L: calculated using the following the formula^21^ from the AERMOD Model Formulation Document^16^.
$$L=\frac{\rho \;c_{p}T_{ref}{u_{*}}^{3}}{kgH}$$
  where *ρ*, *c*_*p*_, *T*_*ref*_, u_*_ and *g* are the same as described above and *k* is von Karman constant (0.4, dimensionless).
- Surface roughness length, z_0_: used HRRR surface roughness variable named “SFC=Ground or water surface; Surface roughness [m]”.
- Bowen ratio, Bo: calculated as the absolute value of the ratio of sensible heat flux (H) to latent heat flux, where the denominator was the HRRR variable “SFC = Ground or water surface; Latent heat net flux [W/m^2^]”. Note, in observations where the latent heat flux was zero and would result in an infinite ratio value, the location’s 2019 estimated median Bowen ratio was used instead.
- Albedo, r (*ϕ*): set to 1 during nighttime and calculated during daytime as the ratio of reflected to incoming shortwave radiation. For daytime hours (i.e., when the beam downward solar flux was positive), albedo was calculated as the difference between downward and upward shortwave radiation, divided by the downward shortwave radiation. The HRRR variables used in this calculation were “SFC = Ground or water surface; Downward short-wave radiation flux [W/m^2^]” and “SFC = Ground or water surface; Upward short-wave radiation flux [W/m^2^]”.
- Wind speed: used the HRRR variable “10[m] HTGL=Specified height level above ground; Wind speed [m/s]”.
- Wind direction in degrees: estimated using u-wind (eastward wind) and v-wind (northward wind) based on the following function:
$$WD=\text{mod}\left(180+\frac{180}{\pi }\text{arctan}2\left({\text{wind}}_{u},\;{\text{wind}}_{v}\right),360\right)$$
  where *wind*_*u*_ is the HRRR variable “10[m] HTGL=Specified height level above ground; u-component of wind [m/s]”, *wind*_*v*_ is the HRRR variable “10[m] HTGL=Specified height level above ground; v-component of wind [m/s]”, and π equals 3.14. *mod*(, 360) is a function to return reminder, which can ensure the results ranges between 0 and 360.
- Height at which the wind was measured: set to 10 meters as we used this height’s wind variables in HRRR.
- Temperature: HRRR temperature variable “2[m] HTGL=Specified height level above ground; Temperature [C]” converted to Kelvin by adding 273.15.
- Height at which the temperature was measured: set to 2 meters as we used this height’s temperature variables in HRRR.
- Precipitation type code (0=none, 11=liquid, 22=frozen, 99=missing): assigned a value of 11 (liquid) to all records for simplicity in data preparation as R-LINE does not use precipitation type in its dispersion calculations.
- Precipitation amount: used HRRR 1-hour forecast precipitation variable “SFC=Ground or water surface; 01 hr Total precipitation [kg/(m^2)]”.
- Relative humidity (%): used HRRR variable “2[m] HTGL=Specified height level above ground; Relative humidity [%]”.
- Station pressure: divided the HRRR variable “SFC=Ground or water surface; Pressure [Pa]” by 10 to convert the unit compatible with AERMET.
- Cloud cover: converted the HRRR variable “EATM=Entire Atmosphere; Total cloud cover [%]” to AERMET decile format for cloud cover (i.e., 0–10 where 5 translates to 50% cloud cover) by dividing the percentage value by 10 and rounding down to the nearest whole number using the floor function.
- Wind speed adjustment flag for adjustment of Automated Surface Observing System (ASOS) wind speed data (ADJ = adjust, NAD = not adjusted): wind speeds, collected at ASOS sites, are truncated rather than rounded to whole numbers. Because this introduces an underestimation bias in wind speeds, AERMET adds ½ knot (0.26 m/s) to all ASOS-based wind speeds to compensate. Because we did not use data from ASOS, this variable was set to “NAD”.
- SUBflag: set to “NoSubs”.

In Scenario 2, we applied upper and lower limits to several meteorological variables derived from HRRR data. These bounds were informed by the AERMOD Model Formulation Document^16^ and were intended to prevent implausible values and unrealistic dispersion estimates. Values below the lower bound were set to the lower limit and those above the upper bound were set to the upper limit. Specifically, sensible heat flux (H) was constrained to −64 to 800 W/m^2^; latent heat flux was constrained to −100 to 800 W/m^2^, and surface friction velocity (u*) and surface roughness length (z_0_) were each constrained to range from 0 and 2. Mixing height (Z_ic_) was capped at 4000 meters. These revised variables were then used to calculate the convective velocity scale (w) and the Monin-Obukhov length (L), which were themselves constrained to ranges of 0 to 2 and −8888 to 8888, respectively. Bowen ratio was limited to a range of −10 to 10.

In Scenario 3, we refined meteorological input processing by calculating sensible heat flux (H) using two different methods based on CBL and SBL. Daytime (CBL) and nighttime (SBL) were identified using the Visible Beam Downward Solar Flux variable, with values greater than zero indicating daytime. For CBL conditions, H was recalculated based on an energy balance theory describing the urban heat island,^22^ as used by the AERMOD manual.^16^ In the theory, H is calculated using the residual net radiation after accounting for ground and latent heat fluxes:

H=netradiation-groundheatflux-latentheatflux

Net radiation was calculated as the sum of downward shortwave and longwave radiation minus the sum of upward shortwave and longwave radiation. These variables are available in HRRR. The HRRR variable “SFC=Ground or water surface; Ground heat flux [W/(m^2)]” was used for ground heat flux while “SFC=Ground or water surface; Latent heat net flux [W/(m^2)]” was used for latent heat flux.

In daytime (CBL) hours, Monin-Obukhov length (L) and convective velocity scale (w) were recalculated with revised sensible heat flux (H), accordingly. In nighttime (SBL) hours, the original values from Scenario 2 were retained. All recalculated variables remained subject to the same physical bounds applied in Scenario 2 to prevent extreme values.

### Observation-based AERMET data

We obtained AERMET surface meteorological data from U.S. EPA. Those data were prepared using measurements from weather stations including Automated Surface/Weather Observing Systems (ASOS/AWOS) and the Aviation Weather Center. The full dataset included data files for 793 stations spanning the continental United States. Data processing was conducted using AERMET version 19191. The observation-based AERMET data served as the reference in subsequent analyses to evaluate the performance of the HRRR-based surface meteorological inputs generated under the three scenarios.

### Case study: application in traffic-related air pollution modeling

To evaluate the performance of HRRR-based AERMET surface meteorological data in comparison to traditional observational data, we operated R-LINE using the four versions of surface meteorological data (three HRRR scenarios and observational data) and test their performance in predicting daily NO_2_ concentrations at EPA Air Quality System (AQS) monitoring sites.

R-LINE is a Gaussian dispersion model designed to estimate ambient air pollution concentrations contributed exclusively by traffic. It uses AERMET surface meteorology data to estimate the dispersed air pollution from traffic. R-LINE (v1.2) software was downloaded from the Community Modeling and Analysis System (CMAS) Center website. Traffic emission inputs for R-LINE were prepared using the methods developed in our previous work^6, 23^ and described briefly in the following paragraph.

For each EPA AQS NO_2_ monitoring site, we extracted road segments within a 10-km buffer from the 2019 roadway inventory shapefile published by the U.S. Department of Transportation’s Highway Performance Monitoring System (HPMS). This shapefile included the geographic locations of major roads along with annual average daily traffic (AADT) counts for each segment. The shapefile also provides AADT for single-unit trucks and combination-unit trucks. We then broke down the total AADT by three vehicle types (single-unit trucks, combination-unit trucks, and others) calculated as the total AADT minus the two truck classes. Emission factors (in g/km per vehicle) were estimated using the MOVES model, which provides emission factors for 13 vehicle classes, five road types, and five fuel types. Factors were generated by month to account for the influence of temperature and humidity on engine efficiency.

Road-specific emissions were then estimated by multiplying road-specific AADT by the corresponding emission rates estimated in the MOVES model. These values were converted to grams of NO_2_ per meter per second to ensure compatibility with R-LINE, which outputs concentrations in μg/m^3^. To minimize the influence of differences in temperature and pressure between the HRRR-based and observational meteorological inputs and to focus on their dispersion modeling performance, we converted R-LINE predictions from μg/m^3^ to parts per billion (ppb) by simply multiplying the values by 0.53, assuming standard temperature of 25°C and 1 atm pressure. Given the meteorological inputs were hourly, R-LINE outputs hourly predictions of traffic-related NO_2_. We aggregated hourly predictions to daily estimates by taking an average of all hours in each day.

We linked daily average R-LINE predictions to daily NO_2_ measurements by matching monitoring site locations and dates. We evaluated the performance of R-LINE using two statistical metrics: the coefficient of determination (R^2^) from linear regression fitted with predicted and measured NO_2_ concentrations and Index of Agreement (IOA).^24^ Assessments were stratified by the distance (<2km, 2–10km, 10–20km, 20–35km, ≥35km) between a given NO_2_ monitoring site and its nearest weather station. For monitoring sites with a weather station closer than 2km (approximating the maximum HRRR grid-to-site distance of 2,121 m), comparison of R-LINE predictions against weather station data allowed for us to evaluate the relative quality of HRRR data. For more distant sites, comparisons allowed for evaluation of whether the 3-km HRRR meteorological data performs better than observational data in areas far from weather stations.

Because site-specific R^2^ values can be strongly influenced by sample size, NO_2_ concentration levels, and whether local traffic is a major contributor to NO_2_ levels, R^2^ alone is not ideal for comparing R-LINE predictions across sites.^25^ Thus, we also calculated the site-specific IOA and estimated the relative difference (%) in IOA between HRRR data and the observational data.

We further conducted linear regression analyses to investigate the factors affecting the IOAs and the relative difference in IOA. Considered factors included percent of land use types (high-density development, low/medium-density development, forest, agricultural land) within 10 km of site, geographic location (NO_2_ site latitude and longitude), distance to nearest weather station, average and standard deviation of elevation within 10 km of site, median AADT and the annual average NO_2_ concentrations at site. Land-use types were derived from the 2019 National Land Cover Database (NLCD)^26, 27^ for a 10-km buffer around each NO_2_ site. The NLCD provides land cover classification at 30-m resolution, with each pixel assigned to one of 16 land cover categories. For 10-km buffer, we calculated percentage of land covered by high-density development defined using the “Developed, High Intensity (code 24)”, percentage of low/medium-density development combined the “Developed, Low Intensity (code 22)” and “Developed, Medium Intensity (code 23)” land use types, percentage of forest defined as the combined “Deciduous Forest (code 41)”, “Evergreen Forest (code 42)”, and “Mixed Forest (code 43)” land use types, and agricultural land combined “Pasture/Hay (code 81)” and “Cultivated Crops (code 82)” land use types.

HRRR variable computations were prepared in R software and executed in batch on the High-Performance Computing system in the Data Science Core of the Dan L Duncan Institute for Clinical and Translational Research at Baylor College of Medicine. Statistical analyses were conducted in R 4.3.3 software. Site-specific results were illustrated in qGIS 3.36.1 software.

## Results and Discussion

**Figure 1.**
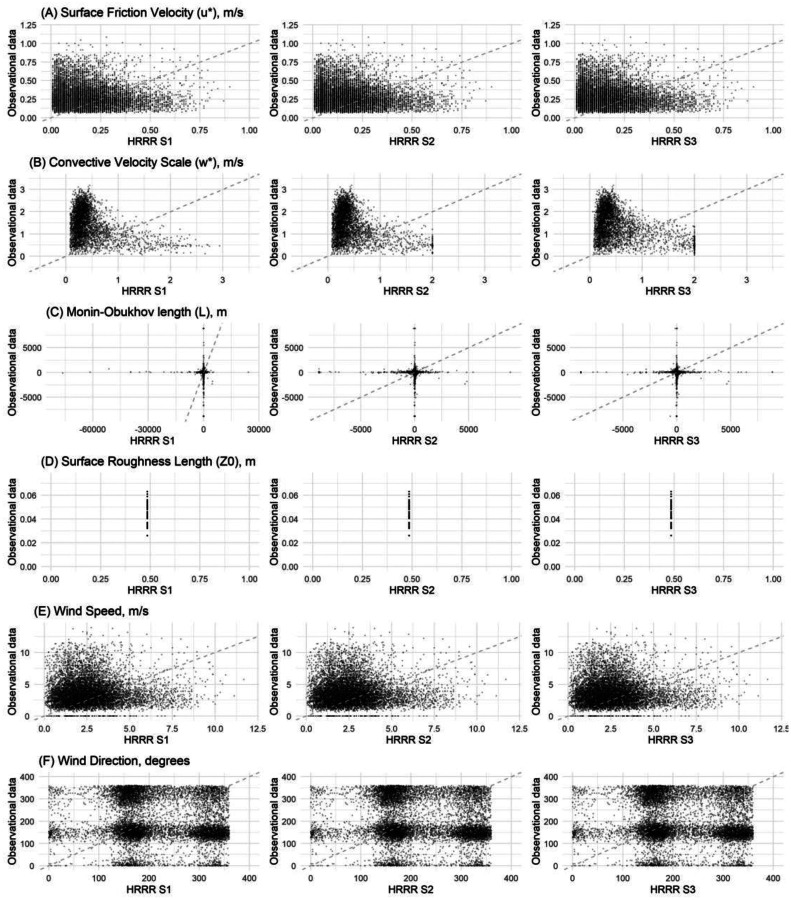
Scatter plots of five R-LINE surface variables across three scenarios of HRRR and observational-based AERMET data. The dashed line (x = y) serves as a reference.

In 2019, there were 446 AQS NO_2_ monitoring sites across the continental United States. Of these, 443 sites were successfully linked to observation-based AERMET data and three were excluded because no observation-based AERMET data were available within the same county. Figure 1 contains scatterplots comparing three HRRR scenarios to observational data for five R-LINE surface meteorological variables. To ensure comparability, results from analyses depicted in Figure 1 were restricted to stations located within 2,000 m of an NO_2_ monitoring site. With this restriction, 13 sites were included.

The scatter plots in Figure 1 indicate substantial differences between HRRR data and observational data, with Pearson correlation coefficients for all comparisons between −0.2 and −0.05. Overall, HRRR tends to underestimate u*, w*, and wind speed at higher values, while overestimating them at lower values. Because the majority of scatter points fall above the x = y reference line, most meteorological data were underestimated by HRRR.

For wind speed, the lower bound in the observational data was smaller than that in HRRR, even after applying a 0.26 m/s adjustment to the ASOS wind data to compensate for low-wind bias. Among all variables analyzed using Pearson correlation, L showed the weakest correlation (r = −0.05), indicating no apparent relationship between the two datasets. This is likely because the sign of L in AERMET is consistently positive during CBL/daytime and negative during SBL/nighttime conditions. In HRRR, although most values follow this pattern, L could still be negative during the day or positive at night. Z_0_ was identical in HRRR across all the 13 near-station NO_2_ sites but varied in the observational data. This is due to the differences in how HRRR and AERMET derive this variable.

Table 1 presents statistics and linear regression results for comparing the NO_2_ measurements and R-LINE NO_2_ predictions using the three HRRR-based scenarios and the station-specific AERMET inputs. Across all meteorology inputs, the average predicted NO_2_ concentrations (6.86 ppb for HRRR and 1.53 ppb for observational meteorology data) were lower than the measured values (8.14 ppb). This result is expected, as R-LINE was run using only traffic-related emissions.

Despite the lower concentrations on average, the linear regression slopes for NO_2_ predictions (x) from all meteorological inputs compared to NO_2_ measurements (y) were less than 1, indicating an overall overestimation. This pattern is likely driven by extreme high predictions in SBL conditions; when L is negative and wind speed is low, R-LINE can generate unrealistically large predictions. Such effects were mitigated when the hourly predictions were averaged to the daily level.

When comparing meteorological inputs, the HRRR-based scenarios produced higher average NO_2_ estimates than did the observational data. The linear regression R^2^ values for the HRRR scenarios were also higher, indicating better overall agreement with NO_2_ measurements. However, the regression slopes for the HRRR data were lower than those for the observational data, suggesting that predictions generated with HRRR inputs are more prone to extreme values. We attribute this to the lower estimates of u*, w*, and wind speed, along with greater variability in L in the HRRR data, which likely produced more stable atmospheric conditions and, in results, led R-LINE to predict higher pollutant concentrations.

**Figure 2.**
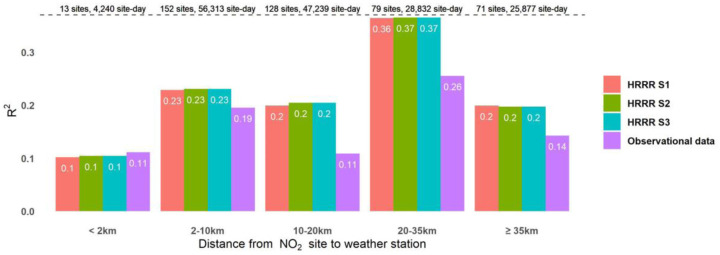
Performance of R-LINE NO_2_ predictions using four types of meteorological inputs across distance categories. R^2^ (y-axis) was estimated using simple linear regression between measured and predicted NO_2_ concentrations.

Figure 2 presents the R^2^ values from linear regressions comparing daily R-LINE predictions to measured NO_2_ concentrations, stratified by the distance between each NO_2_ monitoring site and its linked weather station. For sites located within 2 km of their linked weather station, HRRR-based meteorological data (Scenarios 1–3) had slightly lower R^2^ values than did the observation-based AERMET data. This suggests that the HRRR data may have lower quality than the observation data due to HRRR being subject to modeling errors during its generation. For sites located more than 2 km from their linked weather station, the HRRR data had higher R^2^ values than did the observational data. This finding indicates that the spatially resolved HRRR data better represents local meteorology conditions for those NO_2_ sites without nearby weather stations.

Across all meteorological data sources tested, we did not observe an expected consistent trend of decreasing R^2^ with increasing distance between NO_2_ monitoring sites and weather stations. Among the sites located within 2 km of a weather station, all four meteorological data yielded relatively low R^2^ values (~0.10). This low R^2^ might be due to the influence of aircraft emissions, given most weather stations were located at airports and R-LINE only modeled traffic-related NO_2_. This could result in greater differences between predictions and measurements at these sites compared to others.

The R^2^ values were highest for the “20–35 km” distance category across all four meteorological inputs, because NO_2_ concentrations at these sites had a greater contribution from traffic compared with other subgroups. There was a notable drop in R^2^ observed between the“20–35 km” and “≥35 km” distance categories, for all meteorological inputs. Upon further examination of the “≥35 km” group, we found that 25 out of the 71 sites in this subgroup were in Utah and Wyoming, accounting for nearly half of all NO_2_ monitoring sites in these two states. This concentration in mountainous regions suggests that the reduced predictive performance may be attributed to the complex topography and high meteorological variability in these areas, which may not be adequately captured by either HRRR data or traditional weather station measurements.

**Figure 3.**
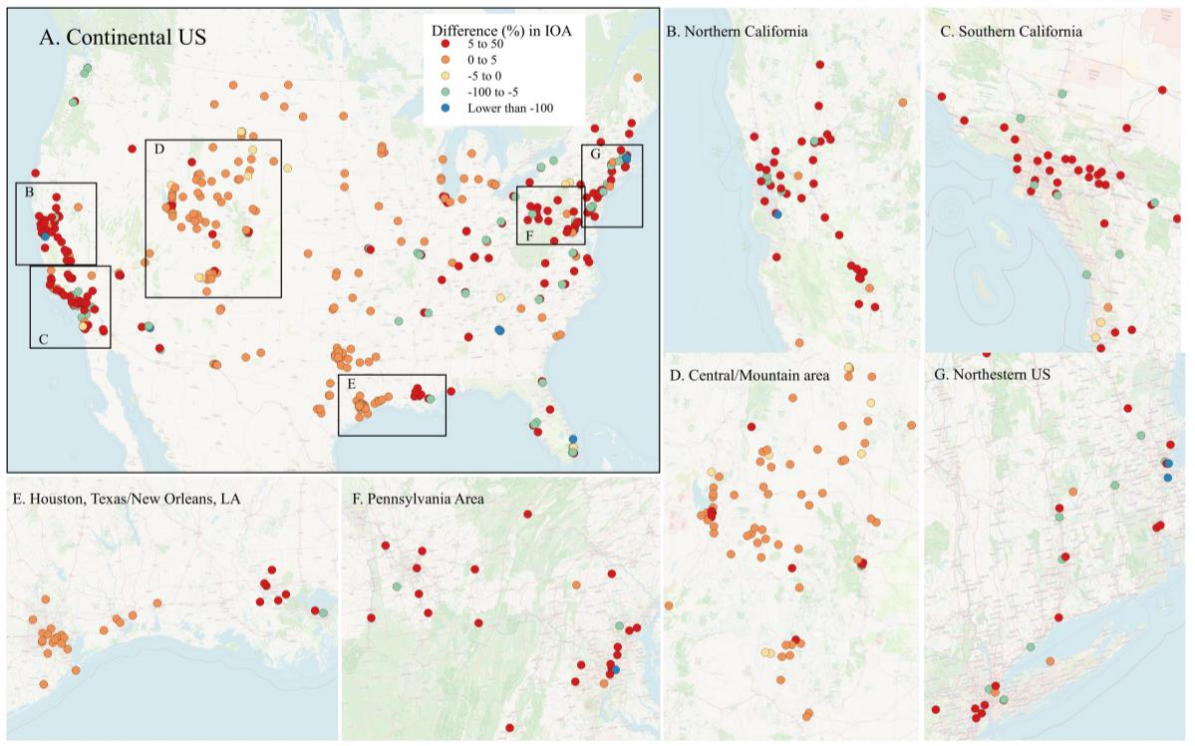
Site-specific differences (%) estimated as 100*(IOA of HRRR S3 – IOA of observational data)/IOA of HRRR S3.

Figure 3 presents site-specific differences (%) in IOA of NO_2_ predictions between the HRRR S3 data and observational data across the continental US as well as areas with high density of NO_2_ sites. The differences between the two sets of results were relatively smaller in the Midwest. The difference between the two sets of results were highly related to the data quality of HRRR. To our knowledge, only one prior study^28^ has investigated biases in dispersion-related variables from HRRR, focusing on the southwestern United States. That study found that HRRR wind speed data were subject to more errors over forested and mountainous terrain, likely due to the absence of observational data in these areas to inform the HRRR model. As shown in Figure 3, we did not observe such pattern. However, the traffic emissions and the distance between NO_2_ sites and nearest weather station can also affect the quality of predictions (Figure 2).

Table 2 presents the factors significantly associated with site-specific IOAs for HRRR Scenario 3, observational data, and their relative IOA differences. The direction of the coefficients indicates how local characteristics influence model performance. IOA was higher at sites located in low- to medium-density development areas, which are most commonly associated with single-family housing units. In contrast, IOA was lower at sites located in high-density development, such as apartment complexes, row houses, and commercial/industrial areas. And for HRRR S3, IOA varied with geographic location, with higher values observed at sites further north (higher latitude) or further west (lower longitude). In the IOA difference model, longitude was negatively associated with relative performance, indicating that HRRR outperformed observational data at a greater degree at western sites compared to eastern sites. Finally, sites with higher NO_2_ concentrations showed worse performance of HRRR compared to observational data. Together with the finding that high-density development area is a negative predictor of IOAs, we hypothesized that high-NO_2_ sites are typically located in urban or near-urban settings, where meteorological factors (e.g., wind speed, mixing height, turbulence) are affected by urban features, such as tall buildings or urban heat islands. Such microscale effects are difficult to capture with either HRRR’s gridded meteorology or standard observational data. However, observational data may still had better correlations with local meteorology than modeled HRRR data in urbans.

Our evaluation demonstrated several advantages of using HRRR as the meteorological input source for R-LINE, including improved representation of local meteorological conditions and less biased daily NO_2_ predictions compared with traditional station-specific observational data. Although the increase of linear regression R^2^ was not substantially higher by using HRRR compared using observational data, the difference remain represents a relative improvement of over 60% in explained variance, which is an important gain in R-LINE predictions. Moreover, HRRR data are ready-to-use and eliminate the need to run complex meteorological models such as MM5 or WRF.

Using HRRR to operate dispersion models presented several limitations. First, some key variables required by AERMET, such as mixing height and vertical gradients of potential temperature, are not directly available from HRRR. In our framework, these missing variables were either approximated using a proxy variable or assigned a fixed value. Future work should focus on improving methods to estimate these parameters from HRRR’s layer-specific products. Second, HRRR-based meteorology can lead to more extreme high predictions in R-LINE, largely due to the occurrence of numerous negative L values during daytime hours that triggered the SBL status. Thus, we do not recommend using raw, hourly HRRR-based dispersion model outputs as direct estimates of pollutant concentrations. Instead, daily or longer-term averages should be used to mitigate these extremes.

In summary, we developed a novel, low-effort framework of preparing surface meteorological data suitable for use in dispersion models AERMOD and R-LINE, leveraging the spatially resolved HRRR dataset. Both AERMOD and R-LINE have been widely used in multiple regulatory capacities. Further, the two models have been increasingly used in air pollution modeling and epidemiologic research. Our methods will greatly improve these models’ predictions in air pollution modeling, especially in areas lacking nearby observational weather data.

## Figures and Tables

**Table 1.** Summary of Daily NO_2_ Measurements and R-LINE Predictions Using Different Meteorological Inputs at 443 Monitoring Sites in 2019.

	Site-day	Mean NO_2_, ppb (SD)	Equation^a^	R^2^^b^
EPA measurements	163,084	8.14 (7.23)	-	
HRRR Scenario 1	163,084	6.86 (14.9)	y=0.24 x +6.41	0.257
HRRR Scenario 2	163,084	6.86 (14.9)	y=0.24 x +6.41	0.257
HRRR Scenario 3	163,084	6.86 (14.8)	y=0.25 x +6.36	0.261
Observational data	163,023^c^	1.53 (4.05)	y=0.71 x +7.02	0.162

a In the equation, y=measured NO_2_ concentrations (ppb) and x=R-LINE predicted NO_2_ concentrations (ppb).

b R^2^ was obtained from the linear regression listed on the left.

c There are missing days in weather station meteorology data.

**Table 2.** Significant predictors of index of agreement (IOA) for HRRR-based and observational meteorological inputs, and their relative differences.

Dependent variable^a^	Independent variables with p-value <0.05	Coefficient^c^
HRRR S3 IOA	% high-density development	−0.003
	% low/medium-density development	0.001
	Latitude in degree	0.003
	Longitude in degree	−0.001
	Average elevation (km)	−0.079
Observational data IOA	% high-density development	−0.003
	% low/medium-density development	0.001
	% agricultural land	−0.001
	Average elevation (km)	−0.038
Differences (%)^b^ in IOA	Longitude in degree	−0.336
	Average NO_2_ (ppb) at site	−1.047

a. Each linear regression model was adjusted with high-density development, low/medium-density development, forest, agricultural land) within 10 km of site, geographic location (NO_2_ site latitude and longitude), distance to nearest weather station, average and standard deviation of elevation within 10 km of site, median AADT and the annual average NO_2_ concentrations at site as independent variables.

b. Site-specific differences (%) estimated as 100*(IOA of HRRR S3 – IOA of observational data)/IOA of HRRR S3, same as displayed in Figure 3.

c. For the Differences (%) in IOA model, a positive coefficient indicates that HRRR S3 outperformed observational data, while a negative coefficient indicates the opposite.

## Data Availability

The R scripts for generating HRRR scenarios are publicly accessible via a GitHub repository (https://github.com/zxy1219/HRRR_surface_met_data).
